# HoloLens^®^ platform for healthcare professionals simulation training, teaching, and its urological applications: an up-to-date review

**DOI:** 10.1177/17562872241297554

**Published:** 2024-12-08

**Authors:** Octavian Sabin Tătaru, Matteo Ferro, Michele Marchioni, Alessandro Veccia, Oana Coman, Francesco Lasorsa, Antonio Brescia, Felice Crocetto, Biagio Barone, Michele Catellani, Alexandra Lazar, Marius Petrisor, Mihai Dorin Vartolomei, Giuseppe Lucarelli, Alessandro Antonelli, Luigi Schips, Riccardo Autorino, Bernardo Rocco, Leonard Azamfirei

**Affiliations:** Department of Simulation Applied in Medicine, George Emil Palade University of Medicine, Pharmacy, Science, and Technology of Targu Mures, Targu Mures, Romania; Istituto Europeo di Oncologia, IRCCS—Istituto di Ricovero e Cura a Carattere Scientifico, via Ripamonti 435 Milano, Italy; Università degli Studi di Milano, Milan, Italy; Department of Medical, Oral and Biotechnological Sciences, G. d’Annunzio, University of Chieti, Urology Unit, “SS. Annunziata” Hospital, Chieti, Italy; Department of Urology, ASL Abruzzo 2, Chieti, Italy; Department of Urology, University of Verona, Azienda Ospedaliera Universitaria Integrata of Verona, Verona, Italy; Department of Simulation Applied in Medicine, George Emil Palade University of Medicine, Pharmacy, Science, and Technology of Targu Mures, Targu Mures, Romania; Department of Emergency and Organ Transplantation, Urology, Andrology and Kidney Transplantation Unit, University of Bari, Bari, Italy; Department of Urology, European Institute of Oncology, IRCCS, Milan, Italy; Università degli Studi di Milano, Milan, Italy; Department of Neurosciences and Reproductive Sciences and Odontostomatology, University of Naples Federico II, Naples, Italy; Department of Surgical Sciences, Urology Unit, AORN Sant’Anna e San Sebastiano, Caserta, Italy; Department of Urology, European Institute of Oncology, IRCCS, Milan, Italy; Università degli Studi di Milano, Milan, Italy; Department of Anesthesia and Intensive Care, George Emil Palade University of Medicine, Pharmacy, Science, and Technology of Targu Mures, Targu Mures, Romania; Department of Simulation Applied in Medicine, George Emil Palade University of Medicine, Pharmacy, Science, and Technology of Targu Mures, Targu Mures, Romania; Department of Urology, Medical University of Wien, Wien, Austria; Department of Emergency and Organ Transplantation, Urology, Andrology and Kidney Transplantation Unit, University of Bari, Bari, Italy; Department of Urology, Azienda Ospedaliera Universitaria Integrata of Verona, University of Verona, Verona, Italy; Department of Medical, Oral and Biotechnological Sciences, G. d’Annunzio, University of Chieti, Urology Unit, “SS. Annunziata” Hospital, Chieti, Italy'; Department of Urology, ASL Abruzzo 2, Chieti, Italy; Department of Urology, Rush University Medical Center, Chicago, IL, USA; Unit of Urology, Department of Health Science, University of Milan, ASST Santi Paolo and Carlo, Milan, Italy; Matteo Ferro is also affiliated to Unit of Urology, Department of Health Science, University of Milan, ASST Santi Paolo and Carlo, Milan, Italy; Bernardo Rocco is also affiliated to U.O.C. Clinica Urologica, Dipartimento Universitario di Medicina e Chirurgia Traslazionale Fondazione Policlinico Universitario, IRCCS, Rome, Italy; Università Cattolica del Sacro Cuore, Milan, Italy; Giuseppe Lucarelli is also affiliated to Department of Precision and Regenerative Medicine and Ionian Area Urology, Andrology and Kidney Transplantation Unit, Aldo Moro University of Bari, Bari, Italy; Department of Anesthesia and Intensive Care, George Emil Palade University of Medicine, Pharmacy, Science, and Technology of Targu Mures, Targu Mures, Romania

**Keywords:** augmented, HoloLens®, medicine, mixed reality, teaching, urology, virtual

## Abstract

The advancements of technological devices and software are putting mixed reality in the frontline of teaching medical personnel. The Microsoft^®^ HoloLens 2^®^ offers a unique 3D visualization of a hologram in a physical, real environment and allows the urologists to interact with it. This review provides a state-of-the-art analysis of the applications of the HoloLens^®^ in a medical and healthcare context of teaching through simulation designed for medical students, nurses, residents especially in urology. Our objective has been to perform a comprehensively analysis of the studies in PubMed/Medline database from January 2016 to April 2023. The identified articles that researched Microsoft HoloLens, having description of feasibility and teaching outcomes in medicine with an emphasize in urological healthcare, have been included. The qualitative analysis performed identifies an increasing use of HoloLens in a teaching setting that covers a great area of expertise in medical sciences (anatomy, anatomic pathology, biochemistry, pharmacogenomics, clinical skills, emergency medicine and nurse education, imaging), and above these urology applications (urological procedures and technique, skill improvement, perception of complex renal tumors, accuracy of calyx puncture guidance in percutaneous nephrolithotomy and targeted biopsy of the prostate) can mostly benefit from it. The future potential of HoloLens technology in teaching is immense. So far, studies have focused on feasibility, applicability, perception, comparisons with traditional methods, and limitations. Moving forward, research should also prioritize the development of applications specifically for urology. This will require validation of needs and the creation of adequate protocols to standardize future research efforts.

## Graphical abstract


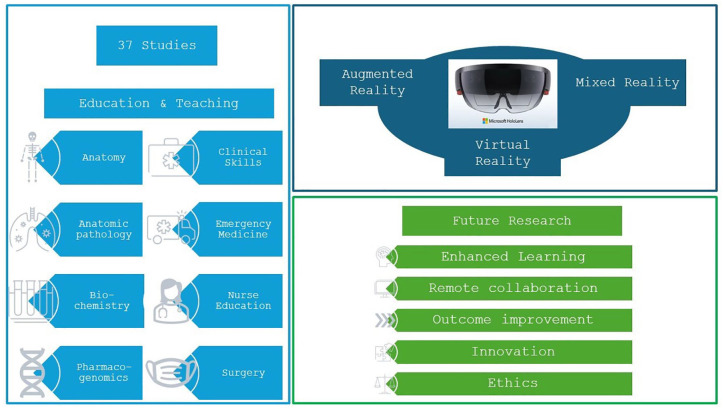


## Introduction

The pinnacle of the digital revolution is bringing the artificial environments in medicine, highlighting the endless opportunities for training, teaching, and learning, in medical and surgical fields, to new higher grounds. Technical and hardware advancements are today able to allow new peaks in digital simulation with the new virtual reality (VR), augmented reality (AR), and mixed reality (MR) technologies, such as the Microsoft^®^ HoloLens platform.^1^ The possibility for endless repetitions and feedback receiving for different skill acquisition surpasses the traditional learning methods. A brief description of digital reality with a focus on the HoloLens^®^ platform will allow healthcare professionals to immerse themselves in the digital revolution of simulation.

### Virtual, augmented, and mixed reality

Due to the advancements in technology, VR, AR, and MR are currently seen as different modalities suitable for healthcare professional training and that will allow students to gain medical knowledge in a virtual world through the use of images, sounds, smells, and other human perceptions.^2,3^ Starting from VR technology, which enables the student to see, hear and manipulate objects, in an environment fully generated by a computer,^4,5^ AR, which enhances the real world in a 3D fashion,^6,7^ and MR, which allows the student to interact with real and digital elements, combining them all, these technologies can improve simulations for teaching purposes.^8,9^ These technologies can provide fast, adapted, and cost-effective simulated learning^3^ and could represent a game changer in traditional cadaver dissection, anatomical, and surgical skills, radiology teaching, and radiotherapy.^10–15^ In emergency medicine, applications of training and learning could represent a forward leap due to the possibility of having all information in the field of view (FOV) and real-time usage.^16,17^ There are multiple platforms that can allow teaching and training in digital reality. We have further focused our research on the Microsoft HoloLens platform, currently the most used in surgical sciences, especially in urology.

### The Microsoft HoloLens

Microsoft HoloLens^18^ was developed in 2016 and offers MR possibilities in 3D vision using a head-mounted display or headset. It enhances senses by projecting 3D holograms in a real environment, without external tracking devices and markers, making it completely self-contained and allowing users to interact with them.^19^ The technical characteristics make the platform have a vast array of built-in sensors (an inertial measurement unit, five visible light cameras for assessing the environment, a time-of-flight depth sensor, an ambient light sensor, and four microphones.^19^

The built-in cameras and depth sensors can map the close surroundings and adapt the holograms to the environment. Improvements in the hardware and software of the HoloLens 2^®^ led to the release of the second generation in 2019.^20,21^ The stereoscopic virtual images are shown on two semitransparent lenses for 3D rendering, combined with the surrounding environment.^22^ The holographic processing unit is putting out enough processing power to smoothly acquire the user’s virtual actions and movements in the real environment through continuous adjustments performed in real-time.^23^ Not being conceived for medical applications, HoloLens presents some intrinsic limitations. Hübner et al.^24^ found that the depth sensor is affected by noise; therefore, the accuracy is reduced. Another limitation is related to the spatial-temporal differences between the two realities (real and VR) to work properly: tracking, registration, and rendering have to work in real time.^25^ Hardware enhancement can somehow limit this, but it will raise the cost and need remote assistance and additional latency. The fixed focus plane at 2 m limits the surgical application because usually surgical procedures are performed closer, reducing HoloLens use when great precision and accuracy are needed, such as surgery itself.^26^

The direction of the headset vision is processed using the head movements allowing the users to interact with the virtual content by gestures and automatically recognized speech.

The future is here for interactive, virtual, augmented, and mixed reality teaching; therefore, the aim of this review is to identify and comprehensively analyze the latest applications of the Microsoft HoloLens for medical teaching purposes. We analyze the timeline of published articles in the last years, the research areas of current interest, and the potential future of simulation for medical healthcare professionals.

## Materials and methods

From January 2016 to April 2023, a comprehensive literature search was performed through PubMed/Medline and Google Scholar databases. The search was limited to articles published in English with available abstracts. The search keywords used were “HoloLens^®^, medicine, simulation, teaching, learning, training, medical students, nurses, residents, anatomy, emergency, imaging, surgery, urology.” The articles identified were also screened to retrieve missed evidence from reference lists. Articles assessing Microsoft HoloLens having for teaching medical purposes (teaching to medical students, residents, young doctors, or highly trained specialists) were deemed eligible. On the contrary, articles reporting incomplete data, abstracts, studies with quantitative or qualitative results, or non-human subjects, books or book chapters, letters, review articles, editorials, and short communications were excluded. Five areas of interest were found as an application of HoloLens as a means of teaching and training of medical students, nurses, novices, trainees, and young specialist doctors. We have further analyzed and discussed the timeline of published articles, area, and the number of research subjects. Tables that follow are incorporating the studies, year of publication, methods, methodology, software used, and results and outcomes. The reporting of this study conforms to the Preferred Reporting Items for Systematic reviews and Meta-Analyses (PRISMA),^27^ and a checklist has been created as a Supplemental File.

## Results

**Figure 1.**
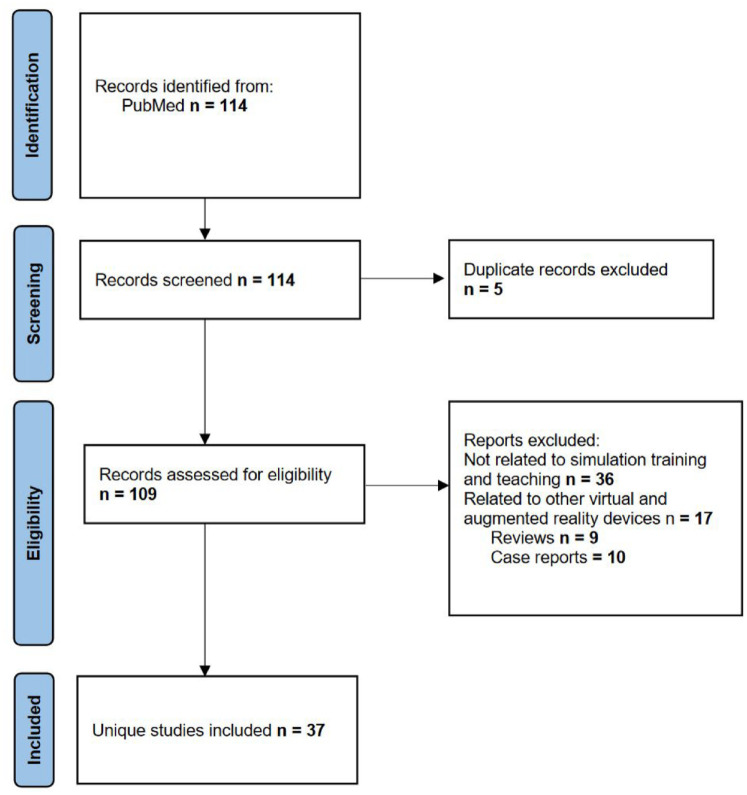
Flow diagram of study selection process depicted by the PRISMA guidelines. PRISMA, Preferred Reporting Items for Systematic reviews and Meta-Analyses.

Overall, 109 articles were identified and assessed for eligibility after duplicate removal and title and abstract reading. Among these, 36 were not related to training or teaching, 17 discussed other types of AR devices, and 9 and 10 were reviews and case reports, respectively. Thirty-seven articles met the criteria for the final analysis (Figure 1).

Since its release in 2016, we have found studies involved in teaching only from 2018, with a steady increase in the following years, with the highest number of articles published in 2021. In 2022 less research has been performed than in 2021, likely caused by the release of the second generation of HoloLens, and most probably researcher shifted their attention to the study of this new hardware. It is clear that the most recent pandemic of coronavirus took a toll on the number of research performed in this time frame. Topics studied imply a vast area in the medical field, starting with surgical and urological interventions, anatomy and anatomical structures teaching, biochemistry and pharmacogenomics, clinical skills emergency medicine and nurse education, and last but with a great future potential is the diagnostic imaging studies. Anatomy, surgery, and emergency medicine seem to have benefited so far from this new technology, due to a high interest in knowledge acquired for teaching purposes, alleviating the surgical teaching curve and probably because of a great simulation teaching and training history in preparing emergency doctors. Our results are listed and discussed below, having in mind the HoloLens teaching and training possibilities in anatomy, anatomic pathology, biochemistry and pharmacogenomics education, improvement of clinical skills, emergency medicine and nurse education, imaging technologies education, and teaching of surgical interventions, especially of urological skills development of healthcare professionals.

### HoloLens in anatomy, anatomic pathology, biochemistry, and pharmacogenomics education of healthcare professionals

Anatomy has been taught at medical schools since ancient times using books, atlases, and human cadavers.^28^ Very little has changed during the years in the way that anatomists are implementing anatomical knowledge to medical students, and this poses great challenges due to a small number of cadaveric specimens, 3D perception of students, a great amount of time used to read, draw figures, repeat, and ultimately understand the human anatomy.^28–30^ Research aimed to define the role of mixed reality in anatomy teaching and also to compare it with traditional methods, and yielded good results.^10^

Stoyanovska et al.^10^ developed a randomized controlled trial (RCT) allowing 64 participants to be assigned to three separate cohorts in order to assess the effectiveness of HoloLens in teaching musculoskeletal anatomy to medical students and to compare it with traditional dissection of cadavers. The authors found no statistical difference between the groups of patients who studied first on MR and then on the traditional method and the second group who studied first the traditional method and after on the MR platform (*p* > 0.05) and a positive correlation between the MR practical examination and cadaver practical examination scores (*r* = 0.74, *p* < 0.01) across all students’ cadaveric dissection. Limitations of the study are the use of small sample size and no recorded data on the additional training time of participants besides the time spent for the RCT. The strength is an RCT design with well-set inclusion and exclusion criteria. It seems that MR has a non-inferior ability to teach anatomy to medical students. Gnanasegaram et al.^31^ assessed the feasibility and effectiveness of learning ear anatomy using HoloLens and a 3D model of the middle and inner ear and analyzed results from 29 medical students. Test scores did not differ between groups in anatomic knowledge, but all groups had improvements (*p* < 0.001), equal across all interventions (*p* = 0.06), and satisfactory rates were higher for HoloLens compared to didactic or computer teaching (*p* < 0.001). The lack of long-term follow-up to evaluate knowledge retention and a loss to follow-up of participants are drawbacks, but a combination of different learning methods seems to lead to long-term data retention. Kumar et al.^32^ to deeply understand the way that facial anatomy could be improved in terms of time spent and to ease medical teaching present a HoloLens model to facilitate anatomy learning for medical students. Validity assessment of a 3D MR model provided by expert participants may highlight new challenges and limitations of MR/AR teaching of anatomy. Limitations arise because the density of polygons in the models and the quality of surface textures limit the level of detail that can be achieved for MR models. Results can validate that the HoloLens platform can have the ability to accurately render 3D images with labels and induce a good perception of the face for the students. Duncan-Vaidya et al.^33^ aimed to identify if AR can be effective and user-friendly for community college students studying the anatomy of the skull through traditional methods or HoloLens. The study only used 32 participants, but randomization can be regarded as a strength. Students were randomly assigned to the traditional group (*n* = 17) or the AR group (*n* = 15) upon enrollment. AR has been identified as an effective tool, equal to traditional methods. Bogomolova et al.^34^ assessed the points of view of perceived learning and assessment of undergraduate and postgraduate students along with expert teachers with the use of HoloLens in an anatomical lesson, using a Likert scale scoring system. Studying only the personal experience with the HoloLens assessment scenario, and even though the overall experience with it was positive and preferred over the traditional methods, limits the quality of the results. Shahbaz et al.^35^ researched the possibility of training and detecting liver diseases with the use of a HoloLens model to preoperatively identify the complex anatomy of blood vessels including 26 clinician experts in liver surgery. The authors have conducted a questionnaire to obtain and analyze results. Having a model of the liver, the authors sent the reconstructed images to the HoloLens. The perceived use in planning, safer paths for surgery, to lower intraoperative complications and training were 100%, 84.6%, 69%, and 84%, respectively. Based on the questionnaire results, MR is a potential alternative for above-mentioned applications. In pathology, Hanna et al.^36^ aimed to research the HoloLens clinical and non-clinical potential (autopsy, 3D visualization of macro and microscopic tissues, navigation for whole-slide-images (WSI), telepathology, and correlation with radiology). Pathology residents worn the HoloLens and were assisted by the experts to annotate and superimpose the radiology findings as well as WSI with ease. Peterson et al.^37^ used HoloLens to reinforce learning to teach bio-molecular structures in a 3D fashion by creating colored custom biomolecules in HoloLens visors and MR exercises to reinforce concepts of biomolecule structures. The study design has poor relevance in terms of differentiating from traditional methods. Roosan et al.^38^ aimed to model pharmacogenomics education using the HoloLens platform, and system usability scale questionnaires-based were applied. Fifteen participants viewed videos for education purposes with text and 3D structures of pharmacogenomics, resulting in a score of 83 and a standard deviation of 6.6, and concluded that the tool could bear potential for precision medicine. Pelanis et al.^39^ investigated if the use of a 3D model of the liver seen in mixed reality with HoloLens performs better than traditional 2D images and found that 82 doctors correctly identified specific lesions faster than using MRI images (*p* < 0.001).

Results from studies show that medical students, specialists, and senior doctors can easily learn anatomical structures with MR as with the traditional methods, MR being at the same time well received and with positive feedback and probably more effective in complex spatial relationships of anatomical landmarks as compared with 2D modalities (textbooks, atlases, and images). By limiting the use of cadaveric dissection, for which there is a growing shortage, also the costs can be lowered by this sustainable approach. Surgically, precise preoperative planning, and accurate intraoperative identification of anatomy, superimposed with radiology images, will increase surgical outcomes, creating a virtual workstation, and adding dynamicity for spatial digital data manipulation. Most studies are small sampled, not grouped and used non-validated questionnaires. These are well-known biases that can influence the obtained results. Therefore, these results are subjective to poor interpretation. Only one design allowed randomization which can raise the quality of the results. The used questionnaires were not validated for the studies purposes, and they only used general items for assessing cognitive effectiveness and satisfaction. Therefore, questionnaires do not objectively validate the appraisal of the effectiveness of MR methods compared or not to the traditional teaching methods limiting further the generalization of results. An interesting result is that also students and teachers are eager to use these new technologically advanced tools in their quest for knowledge, and MR devices-based learning seems to be non-inferior to traditional methods. It is clear that the future is going to be challenging both for students and trainers. Good quality data and better and faster teaching modalities with enhanced retention possibilities will make future great anatomy teaching improvements. Table 1 summarizes the literature evidence for anatomy, anatomic pathology, biochemistry, and pharmacogenomics education with the use of HoloLens.

**Table 1.** Reviewed publications related to the HoloLens headset by methods, methodology, software and results for anatomy, anatomic pathology, biochemistry, and pharmacogenomics education.

Anatomy, anatomic pathology, biochemistry, and pharmacogenomics education						
Article	Year	Aim	Methods	Methodology	Software	Results
Stoyanovska et al.^10^	2020	Effectiveness for teaching musculoskeletal anatomy	MR	MR + cadaver, Cadaver + MR Final evaluation = Cavader + MR	HoloLens + 3ds Max, Autodesk	No statistical difference in the scores of the groups (*p* > 0.05).
Gnanasegaram et al.^31^	2020	Feasibility and effectiveness of learning ear anatomy	AR	Didactic Lecture, web-based Computer Module and Holographic Model	Unity^®^ software, HoloLens	Knowledge improvement not statistical different (*p* = 0.06). Satisfactory rates higher in MR
Kumar et al.^32^	2021	Anatomy face teaching	MR	Close-range photogrammetry Technique, scanning with DSLR cameras, software processing	3DF Zephyr 3.503 software, HoloLens	Validity mean score 4.5 (agree and strongly agree)
Duncan-Vaidya et al.^33^	2021	Skull anatomy teaching	AR	Efficacy of AR acquiring anatomical knowledge	iWorks physiology, HoloLens and Anatomage virtual dissection table softwares	AR equally effective as traditional methods (before and after study) (AR, *p* = 0.001) the traditional methods, *p* = 0.0007)
Bogomolova et al.^34^	2021	Experience and practical use in anatomy teaching	AR	Development of a virtual 3D assessment tool	HoloLens, Dynamic Anatomy^®^ application	Best results: Interaction with the examiner mean Likert scale score 1.00 (±0.00) for experts
Shahbaz et al.^35^	2023	Training, anatomical structures, and detection of liver diseases	MR	MR Navigation in Liver Surgical Anatomy: Learning and Training System	Photoshop CC software 2018, MeshlabV2016.12, Digihuman 3D Reconstruction System	7.7% distinguish the accessory hepatic veins Improvement in identifying depth and spatial relationship of intrahepatic anatomy (92%) The perceived use in planning, safer paths, lower the intraoperative complications and training: 100%, 84.6%, 69%, and 84%, respectively
Peterson et al.^37^	2020	Teaching molecular visualization and enhancing visual literacy	AR	Evaluation of protocols and pedagogical approaches	Holocule app, HoloLens, SketchUp Pro, ketchUp Viewer App	Pros: 65% of responders improved visualization of structures in 14% interactive hands-on Cons: 26% limitations in visualizing, modifying, and interacting with a molecule in AR. 25% slow or difficult usage of the software 18% difficulty of using headset
Roosan et al.^38^	2022	Model a pharmacogenomics education platform	AR	System usability	HoloLens	Good performance score (83 SD ± 6.6)
Pelanis et al.^39^	2020	Spatial 3D understanding of anatomy in MR compared to 2D teaching	MR	Comparison of traditional methods and HoloLens	ITK-Snap, 3D Slicer, HoloLens	MR performs faster (mean 6.00 [1–35] s) in lesion identification than MRI (mean 23.5 [4–138] s). *p* < 0.001)

app, application; AR, augmented reality; DSLR, digital single-lens reflex camera; MR, mixed reality; MRI, magnetic resonance imaging; 2D, two dimensional; 3D, three dimensional.

### HoloLens to improve clinical skills, emergency medicine, and nurse education of healthcare professionals

Advancements in technology are bringing ongoing change and will shape the medical area, teaching, and training of healthcare professionals. Still, training and teaching take place at the patient bed, and well-equipped simulator laboratories are scarce.^40^ AR and MR can limit these disadvantages, by bringing this VR superimposed on the real world, closer to the medical students, which will imply fewer investments in medical simulation labs.^41^ Especially for emergency medicine where it is well known that a key factor for success is the level of expertise,^42^ to improve knowledge and emergency skills, an MR simulation, as a continuous medical training will allow the specialist readiness and preparedness for most of the emergencies. In nurse education, VR, AR, and MR will further add value to knowledge in clinical settings.^43^

Schoeb et al.^44^ aimed to aid the improvement of new clinical skills in students using an MR platform and developed a randomized, single-blinded prospective trial on medical students who were taught to position bladder indwell catheters. They compared results from a total of 164 participants divided into two groups (one having an instructor and the other receiving guidance in the HoloLens system) and identified no statistically significant difference between groups as self-evaluation with the HoloLens having fewer usability scores and in the evaluation of learning outcome students performed better in the MR group (*p* = 0.00). Muangpoon et al.^45^ aimed to improve with HoloLens the ability of medical students to understand, perform and assess results from digital rectal examinations. The visualization of the finger and of the internal organs and tracking of the finger movements have been assessed with the aid of magnetic tracking sensors and pressure sensors and displayed on the MR headset. Questionnaires were applied to 9 experienced clinicians and 10 medical students and identified the usefulness of the platform. Van Gestel et al.^46^ studied, in the emergency scenario, the external ventricular drain placement and aimed to identify the accuracy and learning curve using HoloLens and subsequently developed software through inside-out infrared tracking compared with the freehand technique. The 16 medical students achieved a good performance of external ventricular drain placement (using a modified Kakarla scale) after HoloLens guidance (*p* = 0.005) and not by training (*p* = 0.07). In terms of accuracy, HoloLens guidance students had better outcomes and may significantly improve the learning curve. Putnam et al.^47^ compared traditional methods and HoloLens to identify the potential for use and its feasibility in teaching settings for critical pediatric airway management training assessed by questionnaires. Positive results were achieved in realism, interactivity, and an active learning environment. For improving knowledge, the best areas were found to be anatomy, anaphylaxis, Heimlich maneuver, and foreign body removal (*p* < 0.05). Balian et al.^48^ attempted to study the potential of HoloLens to train healthcare providers in cardiopulmonary resuscitation and assess the quality of chest compressions. Realism (82%), helpful (98%), and 94% willingness to use the MR platform have been the most satisfactory items identified. Suzuki et al.^49^ tested the hypothesis that HoloLens will improve central venous access learning more than just ultrasound. Significant improvements in subjective and objective evaluations have been found (HoloLens-only and ultrasound + HoloLens modality (*p* < 0.05) and ultrasound + HoloLens modality (*p* < 0.05). In conclusion, adding HoloLens improves the teaching potential for central vein access and will aid in alleviating the learning curve. In nursing education curricula, Frost et al.^43^ developed a study to explore HoloLens as an educational platform. They selected 171 nurses who received holographic training on Holopatient and were asked to undertake a visual assessment to identify key clinical issues and potential clinical assessments. In the end, students received a 14-item assessment questionnaire. Overall, the system improved engagement in visual learning, helped to identify knowledge gaps and cues, and to recognize that it will lead to better clinical interpretation and decisions, at the cost of some undesired side effects such as slight dizziness and headaches. A novel modality such as MR being further implemented will for sure enhance student motivation and engagement with learning. Studies performed were set as initial assessment studies of feasibility, accuracy and interactivity. Only one study compared traditional methods and HoloLens and obtained positive results. Visual aids combined with real environment interaction lowers the learning curve of obtainable skills of students, such as understanding, performing, and assessing results and better perception of 3D tasks. At this point, there are few well-designed studies, with control groups, randomization of the participants with clear inclusion and exclusion criteria, with a big number of exposures to HoloLens training sessions and long-term follow-up. Some studies did not use a statistical method to calculate the *p*-value corrections. This further limits the obtained results, but the data are there and must be used in larger and well-controlled trials. Table 2 highlights the literature evidence for clinical skills, emergency medicine, and nurse education using HoloLens.

**Table 2.** Reviewed publications related to the HoloLens headset by methods, methodology, software and results for clinical skills, emergency medicine, and nurse education.

Clinical skills, emergency medicine, and nurse education						
Article	Year	Aim	Methods	Methodology	Software	Results
Schoeb et al.^44^	2020	Bladder catheter placement learning and system usability	MR	Comparison of traditional methods and HoloLens	HoloLens^®^	HoloLens has lower usability scores learning outcomes better in the MR group (*p* = 0.00)
Muangpoon et al.^45^	2020	Adding visualization for DRE	AR	Tracking movements and pressure of the examiner’s finger with HoloLens	Unity game engine and HoloLens^®^	Realism of movements (mean 3.9, standard deviation 1.2) and useful for teaching and learning (finger: mean 4.1, standard deviation 1.1; organs: mean 4.6, standard deviation 0.8)
Van Gestel et al.^46^	2021	External ventricular drainage placement	AR	Comparison of free and technique and HoloLens	Unity Technologies application and HoloLens^®^	Better performance after HoloLens guidance (*p* = 0.005) and compared to traditional training (*p* = 0.07)
Putnam et al.^47^	2021	Assessment of use and feasibility in a setting for critical pediatric airway management training	AR	Comparison of traditional methods and HoloLens	HoloLens^®^	Best knowledge improvement: anatomy, anaphylaxis, Heimlich maneuver and foreign body removal (*p* < 0.05)
Balian et al.^48^	2019	Training in cardiopulmonary resuscitation and assessment quality of chest compressions	AR	Assessment of positive and negative observations of the system	CPReality app, HoloLens^®^	Realism (82%), helpfulness (98%)
Suzuki et al.^49^	2021	Improvements in central venous access	AR	HoloLens is superior to ultrasound	3D model software (Blender, Blender Foundation, Amsterdam, Netherlands), AR platform (Unity, Unity Technologies, San Francisco, CA, USA) and HoloLens^®^	Significant improvements (HoloLens-only and ultrasound + HoloLens modality (*p* < 0.05) and ultrasound + HoloLens modality (*p* < 0.05)
Frost et al.^43^	2020	Feasibility and contribution of MR in education of nursing students	MR	A descriptive evaluation study	HoloLens^®^, Holopatient	100% assistance in learning

app, application; AR, augmented reality; DRE, digital rectal examination; MR, mixed reality.

### HoloLens in imaging education of healthcare professionals

Ultrasound education of medical students, residents, and young specialists has been improved in recent years due to advancements and increases in the availability of devices to be used in patient scanning. The limitations are represented by the 2D images availability, with a short variety of clinical specialties using 3D images in diagnosis, but especially in minimally ultrasound-guided procedures such as endovascular aortic repair,^50^ transthoracic and trans-esophageal echocardiography training,^51^ or ultrasound guidance of vascular punctures.^52^ Advances in AR and MR visual technology, tracking, and registration technologies ease the use of image guidance in clinical or surgical procedures. In radiation therapy, the most important aspect is the correct positioning of the patient, the use of MR technologies can probably improve the process by reducing the alignment time^15^ and radioprotection skills for adequate management of radiology imaging.^53^

Lima et al.^53^ sought to train radiologists in radioprotection using the HoloLens platform. A fluoroscopic system has been used to mimic the radiologist’s management in clinical procedures with HoloLens and to adjust the ceiling shields. Intuitive and relevant and potentially improved radioprotection knowledge were among the benefits of the system, and as a negative appreciation, the difficulty in using HoloLens comes as a drawback. Mahmood et al.^51^ aimed to assess the feasibility of HoloLens for transthoracic and trans-esophageal echocardiography training and created custom tutorials based on MRC to help residents see the senior expert perspective. Ultrasound images were incorporated in a 3D anatomical model for teaching relationships between different anatomical structures and didactic lessons, and live practice has been scheduled for accommodation with the HoloLens system. They concluded that the system will potentially have applications in teaching in the future. von Haxthausen et al.^52^ evaluated the 3D (HoloLens 2) and 2D ultrasound guidance of vascular punctures and compared outcomes. The use of HoloLens 2 shortened the time for completion (28.4% reduction), and no difference in the success of the punctures. Table 3 depicts the literature evidence for imaging in healthcare education using the HoloLens.

**Table 3.** Reviewed publications related to the HoloLens headset by methods, methodology, software and results for imaging in healthcare education.

Imaging and HoloLens in healthcare education						
Article	Year	Aim	Methods	Methodology	Software	Results
Lima et al.^53^	2018	Usefulness evaluation of AR in training of medical professionals	MR	A descriptive evaluation study	Game engine Unity (v. 2021.3.11f1) and Mixed Reality Toolkit (v. 2.8.2)	Improvements in “standard knowledge” to the “accurate” level, limited by handling difficulties
von Haxthausen et al.^52^	2023	3D ultrasonography plus AR for guiding vascular punctures	AR	Simulation of a vascular puncture	Unity 2020.3.7f1 (Unity Technologies, San Francisco, CA, USA)	3D ultrasonography AR mode 28% faster compared to 2D ultrasonography AR No significant differences for success rate of vascular puncture (2D—50% vs 3D—72%)

AR, augmented reality; MR, mixed reality; 2D, two dimensional; 3D, three dimensional.

### HoloLens in teaching purposes education of healthcare professionals

Telementoring or office-based mentoring and teaching,^54–57^ as a teaching modality is another area where AR and MR using HoloLens point an interesting area for improving learning in healthcare. The possibility of presenting, in 3D holographic images, different scenarios of clinical diseases^58–60^ or the ability of a senior expert to guide invasive maneuvers^61^ or to emphasize the role of anatomy importance in a very different way than traditional methods for teaching^60,62^ has a future importance to improve knowledge, confidence, and awareness using the latest technologies in immersive VR, AR and MR.

Glick et al. studied^63^ an interesting and developing area, the telementoring, having in mind the potential of AR to decrease decision time and aid to the first healthcare giving emergency aid, from an experienced physician. Specifically, they researched if telementoring through AI would improve thoracotomy performance and raise self-confidence in medical students. Medical students performed chest thoracotomy in a porcine model. Being split into two groups, inexperienced participants in the study were mentored using HoloLens to perform the procedure, and the other group was independent. Quality performance was found to be higher in the remote HoloLens mentored group and definitely self-confidence was found to be significantly higher (*p* = 0.035). It seems that AR and MR devices could have a future extended role in teaching and supervising medical students, possibly young doctors, to improve performance and raise self-confidence. Wolf et al.^61^ investigated whether AR-based compared with traditional methods of training for extracorporeal membrane oxygenation cannulation will improve outcomes (time spent for the procedure, efficacy, information, information acquisition speed, and stimulation). On 21 medical students, AR allowed fewer errors, but with longer training time (*p* < 0.05). Minty et al.^58^ aimed to demonstrate the validity and feasibility of HoloLens 2 for objective structured clinical examinations for 13 undergraduate students in a prospective assessment of different teaching scenarios obtaining a standard usability scale score of 51.5, and concluded that HoloLens is usable, practical, flexible, and convenient technology in an educational context. Dolega-Dolegowski et al.^62^ sought to develop a holographic model using HoloLens 2 to ease tooth root anatomy learning. The 3D zoomable hologram has a clear advantage for teaching anatomy without restrictions in visualization as in traditional methods, and the questionnaire applied for dentist and dental students revealed good scores in the improvement of endodontic treatment, better visualization of the anatomy, and the HoloLens 2 headset was easy to use. In an RCT, Veer et al.^59^ used HoloLens for student learning for asthmatic disease. RCT with one group, medical students experienced different scenarios of asthma with textbooks, and in the second group, HoloLens was the teaching system. Students completed tests before and after exposure to the lessons, as well as a follow-up test two weeks later to assess message retention. Traditional methods achieved better results for obtaining knowledge compared to HoloLens (*p* < 0.05), and no difference in message retention, but the MR model has been perceived as useful and easy to use. Richards et al.^60^ used HoloLens to implement anatomy laboratory sessions for young and inexperienced osteopathic medical students. This study evaluated indicators or behaviors of student engagement (autonomy and time on task, emotional engagement, and cognitive engagement) by incorporating team-based learning and case-based learning. MR resulted as cost-effective, time-efficient, and effective compared to cadaver dissections. Bala et al.^54^ developed a proof of concept trial using HoloLens 2 to identify the feasibility of telementoring for ward rounds and received positive feedback from medical students as enjoyable, easy to access clinical knowledge, easy to interact with MR system, good quality of hologram information provided, and good interaction with the leading physician. Mill et al.^56^ also used telementoring for ward rounds using HoloLens 2 and live-streamed the information for medical students. They strongly agreed that the overall quality of the teaching session and instructors were excellent, but 32% reported cybersickness. Sivananthan et al.^55^ assessed the feasibility of bedside teaching, using HoloLens, for trainee doctors in the COVID-19 era. After that, they evaluated the results with pre- and post-intervention questionnaires. Results showed that the students strongly agreed that bedside teaching is mandatory for education (median 7, IQR 6–7) and helped for learning (median 6, IQR 5.25–7) and worthwhile (median 6, IQR 5.25–7). For the purpose of achieving a reduction in patient exposure and the use of protective equipment in the COVID-19 outbreak, Levy et al.^64^ used HoloLens 2 in ward rounds resulting in shorter time rounds (94 min versus 137 min; *p* = 0.006) and protective equipment reduced by 50%. Studying geriatric patients, Rafi et al.^57^ utilized HoloLens 2 to explore remote bedside teaching for 30 medical students with interaction with senior experts, lectures on exposure to physical signs and 3D holograms. Besides teaching, students had a higher confidence level using HoloLens 2 elevating its potential for future teaching and training of medical students. Lately, Stackhouse et al.^65^ evaluated and compared knowledge retention and engagement of medical students who were taught clinical case vignette scenarios, training videos, and MR using Microsoft HoloLens 2. Interestingly, knowledge retention using HoloLens 2 was comparable with the other two methods. A higher percentage of the 252 medical students reported enjoyment and engagement for the case vignette scenario (*p* < 0.001) than other teaching methods. Compared to traditional methods, this improves thoracotomy performance and raises self-confidence in medical students, improves outcomes time spent for the procedure, efficacy, information, information acquisition speed, and stimulation, without restrictions in visualization. Some studies indicated that traditional methods achieved better results for obtaining knowledge compared to HoloLens.^59^ These are results worth mentioning from an RCT, with 67 participants, a research design incorporating a proper flow (raised questions, availability of methods to answer questions, and validation of results) of activities. Table 4 illustrates the literature evidence for teaching purposes and education of healthcare professionals in healthcare education using the HoloLens.

**Table 4.** Reviewed publications related to the HoloLens headset by methods, methodology, software, and results for teaching purposes in healthcare education.

Teaching purposes education of healthcare professionals						
Article	Year	Aim	Methods	Methodology	Software	Results
Glick et al.^63^	2021	Telementoring and AR influences on chest thoracotomy performance and self-confidence	AR	Comparative of independent and remote AR guidance	HoloLens^®^	Higher confidence in HoloLens group (*p* = 0.035)
Wolf et al.^61^	2021	Training for extracorporeal membrane oxygenation cannulation could improve outcomes	AR	Comparison of traditional methods and HoloLens	Unity 3D Game Engine (Unity Technologies, San Francisco, CA, USA)	AR gave fewer errors and longer training time (*p* < 0.05).
Minty et al.^58^	2022	Validity and feasibility of HoloLens 2 for objective structured clinical examinations	MR	Prospective assessment of different teaching scenarios	HoloLens^®^	Aims validated by Standard Usability Scale score = 51.5
Dolega-Dolegowski et al.^62^	2022	App for internal anatomy of dental roots	AR	Facilitation of learning process	Autodesk Maya, Unity software, HoloLens^®^	Improvements in endodontic treatment, better visualization of the anatomy (max score of 6)
Veer et al.^59^	2022	Knowledge gain and message retention	MR	Comparison of traditional methods and HoloLens	HoloLens^®^	Traditional methods achieved better results for obtaining knowledge compared to HoloLens (*p* < 0.05)
Richards et al.^60^	2023	Assessing student engagement and commitment to learning	MR	Evaluation of team-based, case-based learning and MR teaching	HoloLens^®^, Holo Anatomy software	MR is cost-effective, time-efficient, and effective compared to cadaver dissections
Mill et al.^56^	2021	Assessing telementoring for ward rounds	AR	Live streamed information for medical students	HoloLens^®^	High overall quality of the teaching sessions Drawback: 32% reported cybersickness
Levy et al.^64^	2021	Value and acceptability of MR headset for ward rounds	MR	Comparison of HoloLens and prior time and protective equipment usage	HoloLens^®^	Shorter ward rounds time—94 min versus 137 min *p* = 0.006
Stackhouse et al.^65^	2023	Evaluation and comparison of clinical case vignette scenarios, training videos and MR knowledge	MR	Formative assessment knowledge attainment and student engagement	HoloLens^®^	Better enjoyment and engagement rate for case vignette (*p* < 0.001)

app, application; AR, augmented reality; MR, mixed reality; 3D, three dimensional.

### HoloLens in surgical interventions teaching of healthcare professionals

Scherl et al.^66^ used HoloLens 1^®^ to assess improvements in planning and technical advances of surgical procedures and to proper measure the accuracy of the technique. During surgery, a 2D and 3D model created with MRI was superimposed onto the patient having parotid surgery. A total percentage of 86% of students considered that HoloLens will play an important role in surgical education, and 80% of them considered that is a feasible option for teaching. It seems that the extensive training will improve the positioning of the patient and the developed navigation system is accurate in measuring the 3D hologram in the HoloLens. Liu et al.^67^ assessed the surgical training on an MR surgical scene, the telementoring provided by a senior expert and the on-site surgeon visualized through HoloLens head-set the virtual scalpel maneuvered by the expert and concluded that this experiment demonstrated the potential to improve the surgical performance of trainees.

Morales-Mojica et al.^68^ aimed to improve visualization and developed an MR interface with the use of HoloLens and images from MRI for planning neurosurgical procedures in order to train and teach surgeons. The planning had a very interesting workflow, including pre-defined regions that were inaccessible to the surgeon so as not to damage the surrounding tissues (e.g., brain, vasculature) and 3D visualization that can be very immersive for the surgeon, especially superimposed on the real surgical field.

Guha et al.^69^ developed a feasibility study to assess HoloLens 2 as a modality to improve technical surgical skills in a prospective randomized feasibility study with 36 medical students and trainees taught to perform an arteriotomy and suture. Students received either a video tutorial of with HoloLens 2 system. The MR arm achieved greater improvement in proficiency as compared to the traditional group (*p* = 0.0076) and better consistency in the progression of acquired skills (*p* = 0.026). Compared to traditional methods of teaching, MR devices have the potential to improve consistency and skills for surgical skills. Tadlock et al.^70^ evaluated the feasibility of HoloLens usage in a randomized audio or MR mentoring of combat casualty wounds. A total of 85% of surgical procedures were performed correctly with no difference in the planning and development of surgery (*p* = 0.21) or how the surgeons implement correctly the surgical procedure (*p* = 0.06). No differences between traditional methods and MR mentoring systems have been detected in this study, and the authors concluded the non-inferiority of HoloLens and its future potential.

### HoloLens in urology teaching of healthcare professionals

Wake et al.,^71^ since 2018, aimed to easily identify anatomic structures and to properly plan the robotic partial nephrectomy. By the creation of a plan from radiological images, 3D and AR models, they demonstrated the plan in a female patient who underwent robotic partial nephrectomy, that the application is safe and feasible and can add value to the surgical decisions. The software used was the Unity 3D software and HoloLens. Amparore et al.^72^ assessed the variety of 3D imaging tools for prostate and kidney cancer surgery. At a peer meeting, the participants were asked to fill out questionnaires with their perceptions on VR, AR, and MR technologies, and the authors found that HoloLens was the best AR and MR technology for surgical planning (50% for prostate and 60% for kidney). Antonelli et al.^73^ in 2019 published an article on HoloLens used for pre-operative planning and assessed the use of holograms for pre-operative planning to evaluate vascular anatomy. Moreover, they assessed the inter-reader variability compared to CT scan and found a higher inter-observer agreement (*k* > 0.6) for anatomical structures and a shorter evaluation time for holographic reconstruction (*p* < 0.0001), concluding that HoloLens can be of valuable use for planning a partial nephrectomy and a better visualization of the renal vasculature.

To evaluate the surgeon’s perceptions of the use of 3D holograms for preoperative planning of complex renal tumors, Checcucci et al.^74^ developed questionnaires employed to attendees. Software used were hyper-accuracy 3D (HA3D™) for image reconstructions and HoloLens for mixed reality environment creation. The scores of perceptions were very positive, and after the presentation and the first-hand experience, 64.4% and 44.4% of the surgeons chose a more selective approach for vasculature clamping.

Al Janabi et al.^75^ evaluated the HoloLens as an alternative to endoscopic monitors in the performance, ergonomics, and iatrogenic complications of ureteroscopy in 72 subjects. The HoloLens eased the time to perform the task (*p* = 0.0011) and OSAT scores (*p* < 0.0001) compared to endoscopic monitors, and students reported positive feedback on the potential role in education (97%) and surgical feasibility (95%). Improving performance in trainees is feasible and can be used as an alternative to traditional methods.

Quesada-Olarte et al.^76^ aimed to use the assistance of extended reality for complex penile surgery with the help of HoloLens and found that this technology is feasible in penile surgical planning flow.

Wang et al.^13^ studied the use of HoloLens’s clinical and accuracy for calyx puncture guidance in percutaneous nephrolithotomy patients and in a control group that underwent ultrasound puncture. The HoloLens puncture time was shorter (*p* < 0.001), had fewer puncture attempts (1.4 ± 0.6 vs 2.2 ± 1.5, *p* = 0.009), had an improved stone clearance rate (*p* = 0.19), and had fewer postoperative complications (*p* = 0.074).

For prostate biopsies, Sparwasser et al.^77^ investigated the usefulness of HoloLens in MRI-assisted targeted biopsy of the prostate compared to systematic biopsy and found that HoloLens targeted biopsy increased the detection rate of prostate cancer (47% vs 19%, *p* < 0.05) compared to systematic biopsy, and found to high feasibility items.

Gadzhiev et al.^78^ assessed the role and utility of HoloLens 2 in laparoscopic partial nephrectomy in a prospective randomized trial with the use of custom-made software in 47 subjects. Patients were randomized to ultrasound and HoloLens 2 controlled vasculature and tumor. Results obtained were in favor of HoloLens 2 for time for kidney vasculature detection and the time from the kidney vasculature detection to the tumor discovery (*p* < 0.001). On the 5-point Likert scale questionnaire high scores were obtained assessing the system utility. Time to renal exposure and tumor detection has been improved by MR devices and maintaining the safety of the procedure.

Li et al.^79^ aimed to assess the surgical skills of urologists performing endoscopic kidney stone surgery by eye gaze measurements and found that expert doctors have more focused gaze patterns, leading to increased safety of the procedures, and the HoloLens could be an objective and non-invasive modality to test the surgical competence. Table 5 illustrates the literature evidence for surgical and urological education of healthcare professionals in healthcare education using the HoloLens. Planning of surgical procedures, the positioning of the patient, and the advances in the navigation system could improve technical surgical skills by allowing fewer calyceal kidney puncture attempts (1.4 ± 0.6 vs 2.2 ± 1.5, *p* = 0.009),^80^ had an improved stone clearance rate (*p* = 0.19), and had fewer postoperative complications (*p* = 0.074).^13^ Improving performance in trainees is feasible and can be used as an alternative to traditional methods that increased the detection rate of prostate cancer,^72^ kidney vasculature detection, and the time from the kidney vasculature detection to the tumor discovery.^74^

**Table 5.** Reviewed publications related to the HoloLens headset by methods, methodology, software, and results for surgical and urological purposes in healthcare education.

Surgical and urological interventions teaching of healthcare professionals						
Article	Year	Aim	Methods	Methodology	Software	Results
Scherl et al.^66^	2023	Assessments of planning and technical advances of surgical procedures	MR	Measurements of the accuracy of the technique	HoloLens^®^, Unity 3D engine (Unity 2018.4.22; Unity Technologies, San Francisco, USA) and Microsoft Mixed Reality Toolkit (MRTK Version 2.2; Microsoft Corporation, Redmond, USA)	A total of 86% of participants assess that MR will play an important role in surgical education and 80% considered that is a feasible option for teaching
Liu et al.^67^	2021	Telementoring assessment of surgical technique	MR	Improvement of surgical technique through MR headset	HoloLens^®^, Vuforia, PTC Inc., USA	Improvements in surgical performance
Al Janabi et al.^75^	2020	Evaluation of performance, ergonomics and iatrogenic complications of ureteroscopy	MR	Comparison between monitor and HoloLens**®** performance	HoloLens^®^	Better time to perform the task (*p* = 0.0011), OSAT scores (*p* < 0.0001) 97% of students pointed a role in education and 95% of surgical feasibility
Morales-Mojica et al.^68^	2021	Improvement of visualization for planning neurosurgical procedures in order to train and teach surgeons	MR	Development of a MR interface with the use of HoloLens^®^ and images from MRI	HoloLens^®^, Unity 3D v5.6	Immersive appreciation of the surgeons
Guha et al.^69^	2023	Assessment of HoloLens 2^®^ for improvement of technical surgical skills	MR	Randomized feasibility study for performance of arteriotomy and suture	Microsoft Dynamics 365 Guides, HoloLens^®^	MR arm better proficiency (*p* = 0.0076) and better consistency in progression of acquired skills (*p* = 0.026)
Gadzhiev et al.^78^	2022	Development of a software for MR anatomic model	MR	Prospective randomized trial of laparoscopic partial nephrectomy to control of vasculature and tumor	Inobitec DICOM Viewer Pro, HoloLens^®^	Time for kidney vasculature detection and the time from the kidney vasculature detection to the tumor discovery (*p* < 0.001)
Tadlock et al.^70^	2022	System for synchronous bidirectional expert MR enabled virtual surgery teaching	MR	Randomization to audio or MR mentoring of combat casualty wounds	HTC-Vive (HTC Corp, Xindian District, Taiwan), HoloLens^®^	A total of 85% of surgical procedures were performed correctly with no difference in planning and development of surgery (*p* = 0.21) or how the surgeons implement correctly the surgical procedure (*p* = 0.06)
Checcucci et al.^74^	2021	Surgeon perceptions on the use of 3D holograms for preoperative planning of complex renal tumors	MR	Questionnaires	HoloLens^®^, hyper-accuracy 3D (HA3D™)	After the presentation and the first-hand experience 64.4% and 44.4% of the surgeons choose a more selective approach for vasculature clamping
Wang et al.^13^	2022	Clinical and accuracy for calyx puncture guidance in percutaneous nephrolithotomy	MR	Comparison of HoloLens and ultrasound guidance	HoloLens^®^	HoloLens puncture time shorter (*p* < 0.001), fewer puncture attempts (1.4 ± 0.6 vs 2.2 ± 1.5, *p* = 0.009) improved stone clearance rate (*p* = 0.19) and fewer postoperative complications (*p* = 0.074)
Sparwasser et al.^77^	2022	Usefulness of HoloLens in MRI-assisted targeted biopsy of the prostate compared to systematic biopsy	MR	Comparison of in MRI-assisted targeted biopsy of the prostate and MR compared to systematic biopsy	Hi-RVS Preirus-System and HoloLens^®^	Increased detection rate of prostate cancer (47% vs 19%, *p* < 0.05) compared to systematic biopsy

AR, augmented reality; DICOM, Digital Imaging and Communications in Medicine; Hi-RVS, High-Resolution Variable System; MR, mixed reality; MRI, magnetic resonance imaging; OSAT, overall satisfaction; 3D, three dimensional.

## Discussions

The MR is one of the latest and novel technologies in 3D visualization developed for teaching, and training in anatomical, anatomic pathology, biochemistry, and pharmacogenomics, for the improvement of clinical skills, emergency medicine, and nurse education. For imaging in medicine and in surgical interventions, especially in urology education and teaching, these AI-driven applications hold promise to facilitate improvements in knowledge acquiring and planning of surgical interventions and nonetheless development of technical skills. By merging virtual and real FOVs, MR can enhance sensory experiences, such as vision and hearing, through holographic 3D images. This integration forms the foundation of how MR can support teaching. The technological advancements have made the HoloLens an integrative platform in an educational setting.^81^ The potential for high accuracy and fidelity of the 3D holograms and 3D fashion teaching of anatomy structures deepen the educational process, and this can be achieved with the use of HoloLens. This is possible by setting different virtual dissection planes using hand gestures, enhancing the relevance of surgical experience. The ready-to-use and easy-to-access information on anatomy overcomes the challenges of cadaver use,^60,82^ and the hands-free use of the device enables students to perform other tasks with their hands (taking notes, additional electronic information search, change scenarios, etc.)^82^ and can improve knowledge scores and spatial awareness.^35,39,83^

When comparing HoloLens with the traditional methods (2D images, text notes, videos), there have been published studies that emphasized the improvements of MR technology HoloLens and showed that MR performs faster and has less usability scores learning outcome,^39^ has better performance in HoloLens guidance,^44^ improves knowledge in anatomy, anaphylaxis, Heimlich maneuver, and foreign body removal,^46^ allows shorter ward rounds time,^64^ gives better time to perform tasks,^75^ calyx puncture time in percutaneous nephrolithotomy is shorter and had fewer puncture attempts, and has improved stone clearance rate and fewer postoperative complications.^13^ Some studies found in fact that traditional methods had better results than HoloLens in knowledge gain and message retention although participants perceived HoloLens as more favorable, enjoyable, and useful,^59^ and another study revealed that case vignette gives better enjoyment and engagement rate compared to MR.^65^ These results are due to the hardware and software limitations in terms of slow speed, not user-friendly applications, difficulties in using the MR headsets, and dizziness or headache in some students, as adverse reactions to the use of MR and probably the lack of orientation in using HoloLens.^43^ Overall, the use of HoloLens is regarded as useful in the anatomy teaching of healthcare professionals by the various anatomy models used for perception and understanding improvement in the teaching sessions. With the improvements in the mentioned limitations, the role of MR and HoloLens will probably be more and more used in anatomical teaching settings.

In surgery teaching applications, HoloLens has the potential advantage, that by increasing the time and bringing various types of exposure for students or residents, to ease the learning curve of surgical procedures.^8^ From the identified studies, most of them aimed to basically assess the proper planning and technical advances of surgical procedures,^66^ telementoring assessment of the surgical technique,^67^ improvement of visualization for planning neurosurgical procedures in order to train and teach surgeons,^68^ assessment of HoloLens 2 for improvement of technical surgical skills,^69^ development of software for MR anatomic model for laparoscopic partial nephrectomy to control of vasculature and tumor,^78^ for system development, and for synchronous bidirectional expert MR-enabled virtual surgery teaching in war casualties.^70^ Being an area of novelty in surgical teaching, the use of MR and HoloLens is limited to determine the feasibility and accuracy of the technique, and the potential improvements to the surgical act to compare the results with the classical or traditional methods of visualization. The surgeons acknowledge that HoloLens is a reliable and time-sparing modality to teach students and assistants, and the image guidance should be further studied to apply the full potential in training of novice healthcare providers, doctors in less accessible or due to economic burdens, to improve their knowledge and performance.

In the urological surgery teaching act, there has been identified research that assesses the surgeons’ perception on the use of 3D holograms for preoperative planning of complex renal tumors,^74^ the evaluation of performance, ergonomics and iatrogenic complications of ureteroscopy, the clinical and accuracy for calyx puncture guidance in percutaneous nephrolithotomy, and the usefulness of HoloLens in MRI-assisted targeted biopsy of the prostate compared to systematic biopsy. HoloLens has potential applications in the field of urolithiasis that are embracing technological advancements, adapted instruments, and methods to perform endourologic treatment procedures.^84^ Numerous areas from the urological surgery field have been explored with the use HoloLens to improve outcomes in healthcare education for students and young specialist to ease the learning curve of interventions, to improve knowledge, to change surgery planning, and to improve patient-related outcomes (Table 6).

**Table 6.** Summary of HoloLens applications in healthcare professional simulation training, teaching, and urological applications.

Application area	Description	Key features	Benefits	Challenges
Simulation training	Use of HoloLens for creating immersive training environments	Real-time 3D visualization, interactive scenarios, remote collaboration	Enhanced learning experience, safe practice environment, ability to simulate rare or complex cases	High initial cost, need for technical support, potential for technology-related distractions
Teaching	Integration of HoloLens^®^ into medical education for teaching anatomy, surgical procedures, and patient care	Interactive anatomical models, step-by-step procedural guides, virtual patient interactions	Improved comprehension of complex concepts, interactive and engaging learning, accessibility to remote education	Learning curve for both instructors and students, varying acceptance levels among educators, technical limitations in some institutions
Surgery	Preoperative planning, intraoperative guidance, and patient education	Detailed anatomical overlays, real-time navigation assistance, patient-specific modelling	Increased precision in surgical procedures, enhanced preoperative planning, better patient understanding of conditions and procedures	Integration with existing medical systems, ensuring accuracy and reliability, data privacy concerns

## Limitations and the future of HoloLens

As a novel technology, there are limitations in the use of HoloLens that were discovered and firstly addressed for the first version, the HoloLens 1, regarding the software and hardware that were upgraded to lower the weight, increase the FOV, enhance the lifespan of the battery, and improve the main and graphic processor.^20^ Although these challenges can be overcome by the enhancement, the head-mounted display comfort, compatibility with surgeon loupes, and cost of development are also drawbacks.^8^ These can explain the variance in the results of studies, using both versions of HoloLens headsets.^85^ User experience can also be affected, especially for VR or AR devices consisting in cyber sickness, blurred vision, and headaches that are partially overcome by the development of MR technologies.^20^ There are also limitations of the applications used in conjunction with the devices, such as image quality for HoloUS^85^ and limitations in visualizing, modifying, and interacting with a molecule in AR.^37^ The low evidence and standardization and protocols limit the validation of HoloLens applications. Registration and tracking abilities of HoloLens were at the center of the research, methodological innovative technologies for different applications are scares, and most of them were proposed in other studies. Just addressing usability, feasibility, and perceptions from users is not enough for these MR technologies to advance, and the need for well-designed studies is high. These will need further studies to improve the use of MR technologies for the benefit of the students and healthcare providers.

As we look toward the future, several emerging trends and technological advancements stand to further augment its impact. The next generation of HoloLens aims to reduce cybersickness, blurred vision, and headaches by improving display technology and refining the ergonomics of the headset. These advancements will ensure a more comfortable and prolonged use experience, crucial for medical professionals during extensive training sessions.^20,23,86^ Additionally, improved graphical capabilities and more sophisticated simulation environments will provide more realistic and immersive training scenarios, enhancing the learning experience.^85,87^ Regarding this trend, we may see more integration of AR and VR within the HoloLens platform, offering a cohesive blend of virtual and real-world elements and enhancing realism and interactivity. Further software developments will likely focus on providing higher resolution and more detailed visualizations of medical images and models, including improved tools for visualization, modification and interactions with complex structures such as anatomical parts. Nevertheless, establishing standardized protocols and conducting rigorous, large-scale studies to validate the efficacy of HoloLens applications is crucial. Such efforts will ensure that the technology meets educational and clinical standards, facilitating broader adoption.^19,64^ Among areas requiring further exploration, it could be included cost reduction strategies, customization, and personalization as well as the possibility of interdisciplinary applications. These premises are related to the necessity of improving the affordability of HoloLens technology while permitting the development of customizable and personalized training modules. Lastly, the long-term outcomes of HoloLens training programs have to be explored via longitudinal studies providing deeper insights into its effectiveness while addressing ethical privacy concerns and ensuring compliance with healthcare regulations.^88^

## Conclusion

This review underscores the transformative potential of HoloLens technology in medical education, particularly for medical students, nurses, young specialists, and residents in urology and other medical/surgical specialties. By synthesizing the latest evidence across various applications—from teaching anatomy and surgical procedures to enhancing diagnostic skills—the review highlights significant strides in feasibility, applicability, perception, and comparison with traditional methods. However, as studies increasingly focus on the development and validation of applications, future efforts must prioritize the standardization of research protocols to ensure robust evaluation and widespread adoption. HoloLens’s ability to offer immersive 3D visualizations and interactive simulations addresses longstanding challenges in medical education, providing learners with hands-on experience in a safe, controlled environment of urological applications (urological procedures and technique, skill improvement, perception of complex renal tumors, accuracy of calyx puncture guidance in percutaneous nephrolithotomy, and targeted biopsy of the prostate). The integration of remote collaboration features also democratizes access to expert guidance, fostering inclusivity and enhancing educational outcomes across diverse learning settings (e.g., simulators with 3D hologram capability). Looking ahead, educators are encouraged to integrate HoloLens into curricula, leveraging its capabilities to personalize learning experiences and cater to different learning styles effectively in areas such as robotics urological interventions (3D visualization of complex renal tumors), minimally invasive surgery (percutaneous nephrolithotomy, retrograde flexible ureteroscopy, miniaturization of devices). Concurrently, ongoing research efforts should concentrate on longitudinal studies to assess the technology’s enduring impact on skill acquisition and clinical performance. Policymakers play a pivotal role in supporting infrastructure development and establishing guidelines to facilitate the responsible integration of augmented reality technologies like HoloLens in medical education. By embracing these opportunities and addressing current limitations through collaborative research and development, stakeholders can collectively advance the field of medical education. This proactive approach not only prepares future healthcare professionals in the urology field to meet the evolving demands of healthcare but also ensures the delivery of high-quality patient care globally.

## Declarations

**Ethics approval and consent to participate:** Not applicable.

**Consent for publication:** Not applicable.

**Availability of data and materials:** Not applicable.
